# OCT changes of idiopathic epiretinal membrane after cataract surgery

**DOI:** 10.1186/s40942-020-00239-8

**Published:** 2020-08-04

**Authors:** Jose Luis Vallejo-Garcia, Mary Romano, Luca Pagano, Alessio Montericcio, Alfredo Borgia, Emanuela Morenghi, Paolo Vinciguerra

**Affiliations:** 1 Humanitas Clinical and Research Center, Humanitas University, Via Manzoni 56, 20089 Rozzano, Milan, 20089 Italy; 2 Multidisciplinary Department of Medical, Surgical and Dental Sciences University of Campania Luigi Vanvitelli, Neaples, Italy; 3 Department of Ophthalmology, Istituto Clinico Humanitas, Via Alessandro Manzoni 56, 20089 Rozzano, MI Italy

**Keywords:** Epiretinal membrane, Cataract surgery, Retina, Peeling

## Abstract

**Background:**

We reviewed our experience in the management of cataract and idiopatic epiretinal membrane surgeries at the Humanitas Research Institute–Milan, Italy- over the past 3 years.

**Methods:**

We conducted a single center retrospective observational case series of patients that underwent sequential cataract and idiopatic epiretinal membrane (ERM) surgeries from 2012–2015 in Humanitas Research Institute. Full data was obtained for 53 eyes of 57 patients. Patients with ERM secondary to uveitis or trauma or associated with simultaneous retinal detachment were excluded. Diabetic retinopathy, glaucoma, age-related macular degeneration, and myopia of more than 6 diopters were exclusion criteria as well.

**Results:**

Cataract surgery was not associated with an ERM stage progression at one month follow up, but caused retinal inflammation that resulted in a significant increase in central macular thickness (CMT), macular volume (MV), central macular edema (CME), IS/OS disruption (IS/OS) and neurosensory detachment (NSD). However, there was no significant change in Best corrected visual acuity (BCVA).

**Conclusion:**

We suggest that patients undergoing cataract surgery in the presence of epiretinal membranes need tight follow up to treat and control eventual macular inflammatory changes and eventual prompt vitrectomy if BCVA is threatened.

## Introduction

Epiretinal membrane (ERM), also commonly known as macular pucker, is an avascular fibrocellular membrane that forms at the vitreoretinal interface, causing visual disfunction and metamorphopsia [1]. In a recent review of the epidemiological studies available, prevalence was 9.1% of the total population [2].

The precise pathophysiology of this clinical entity is not yet known, a proliferation of hyalocytes in the setting of anomalous posterior vitreous detachment and vitreoschisis has been proposed as a possible mechanism in the early development of idiopathic ERMs [3]. The ERM can also be formed secondary to retinal vascular or inflammatory diseases, trauma, tumours and intraocular surgery or inflammation [2].

The association between cataract surgery and ERM and the timing of the surgical procedures is to be determined, whether it is preferable a 2-step-sequential approach or a combined surgery [4–6]. Literature seems to have no clear answer yet, so it is important to analyse morphologically whether or not ERM progression is accelerated by phacoemulsification and if there is a consequence of sequential surgery in BCVA.

The only specific study available on this topic dates back to 2008, were Hayashi found that foveal thickness and macular volume were not significantly influenced by cataract surgery and that the visual acuity was not markedly impaired in the first year following surgery [7].

However, with the recent advances in image resolution and acquisition speed, a newly introduced OCT-based classification was proposed by Govetto et al. [8], shifting the attention to the inner retina anatomy. They described continuous ectopic inner foveal layers (EIFL) as the essential element in their grading scheme, which also was correlated with visual impairment.

We have studied retrospectively the behaviour of the retinal configuration and BCVA in patients with ERM that underwent sequential cataract surgery and consecutive vitrectomy after a month.

## Materials and methods

The aim of the study was to analyse the changes of the epiretinal membrane stage, macular status and BCVA before and after cataract surgery.

We conducted a single center retrospective observational case series of patients that underwent sequential cataract and idiopathic epiretinal membrane surgeries from 2012–2015. Full data was obtained for 53 eyes of 57 patients.

Patients with ERM secondary to uveitis or trauma or associated with simultaneous RD were excluded. Diabetic retinopathy, glaucoma, age-related macular degeneration, and myopia of more than 6 diopters were exclusion criteria as well.

We classified our patients according to Govetto’s ERM classification [8] as follows.

Stage 1 was characterized by the presence of the foveal pit and well-conserved retinal layers.

In stage 2 there was the absence of the foveal pit, stage 3 characterised by the presence of ectopic inner foveal layers, whereas in stage 4 there was also a retinal layers disruption.

The presence of macular cystoid edema (CME), disruption of the inner/outer segment (IS/OS) and neurosensory foveal detachment (NSD) was also recorded.

Patients were examined pre-cataract surgery and one-month post-surgery with full ophthalmological examination including BCVA, slit lamp and fundus examination and SD-OCT (Cirrus HD-OCT; Carl Zeiss, Dublin, California, USA) with protocols analysis of central macular thickness (CMT), macular volume (MV). 11 patients had an extended follow up of 3 months due to delay of the vitrectomy. The following exclusion criteria were adopted: previous intraocular surgery or laser refractive procedure, any ocular or systemic morbidity significantly affecting VA (e.g. central corneal opacity, advanced glaucoma, retina vein occlusion or macular degeneration).

### Statistical method

Data were expressed as mean and standard deviation or number and percentage, as appropriated. The comparison between preoperative and 1-month follow up was done with paired t student test or Wilcoxon test, as appropriated, for continuous variables, and with Mc Nemar test for categorical variables.

The association of the preoperative characteristics with BCVA changes was explored with linear regression, while the association with diabetes was explored with logistic regression analysis.

The significance level was set at 0.05 for all test. Statistical analysis was performed with Stata 15 software (StataCorp. 2017, College Station, TX: StataCorp LLC).

## Results

The 57 eyes of 53 patients included 26 (49.1%) men, with a mean age of 71 ± 5 years, 7 (13.2%) patients had type 2 diabetes without retinopathy.

At baseline they presented with an epiretinal membrane stage 1 in 5 (8.9%) eyes, as stage 2 in 24 (42.9%) eyes, as stage 3 in in 22 (39.3%) eyes and as stage 4 in 5 (8.9%) eyes.

Pre-operative characteristics of the population are reported in Table 1.

**Table 1:** Preoperative population characteristics

N	57
Age (years)	71.6 ± 5.3
BCVA	0.46 ± 0.18
ERM staging	2.48 ± 0.79
1	5 (8.9%)
2	24 (42.9%)
3	22 (39.3%)
4	5 (8.9%)
CMT (µm)	443 ± 79
MV (mm^3^)	11.9 ± 1.4
CME	13 (22.8%)
IS/OS disruption	8 (14.0%)
NSD	3 (5.3%)

Staging of the ERM remained stable after cataract surgery in all patients.

Table 2 reports the changing in visual parameters on month after surgery: there was no significant change in BCVA (p = 0.4605), while a statistically significant worsening of the following parameters was observed CMT, CME, disruption in the IS/OS and NSD, while MV showed a significant increasing.

**Table 2:** Pre and 1-Month post cataract surgery values

	Pre	Post	P
BCVA	0.46 ± 0.18	0.45 ± 0.18	0.4605
CMT (µm)	443 ± 79	486 ± 99	< 0.0001
MV (mm^3^)	11.9 ± 1.4	12.4 ± 1.8	< 0.0001
CME	13 (22.8%)	32 (56.1%)	< 0.0001
IS/OS	8 (14.0%)	16 (28.1%)	0.0047
NSD	3 (5.3%)	9 (15.8%)	0.0143

The difference in 1-month with preoperative BCVA wasn’t associated with any of the preoperative characteristics (Table 3).

**Table 3:** Association with variation of BVCA

	Coefficient (95% CI)	P
Age	−0.001 (−0.023–0.021)	0.906
Diabetes	−0.070 (−0.399–0.259)	0.670
ERM staging	0.058 (−0.092–0.206)	0.448
CMT (µm)	−0.000 (−0.002–0.001)	0.847
MV (mm^3^)	0.015 (−0.068–0.099)	0.713
CME	0.128 (−0.143–0.398)	0.349
IS/OS	0.247 (−0.075–0.570)	0.130
NSD	−0.095 (−0.607–0.417)	0.711

We also analyzed our diabetic patients’ group (7 patients in total), finding no statistically significant difference when compared with the non-diabetic patients. The data are reported in Table 4. This analysis, however, is partially influenced by the low population number.

**Table 4:** Different variables in diabetic and non-diabetic groups

	DM	No DM	OR (95% CI)	P
N eyes	8	49		
Age	72.8 ± 8.2	71.4 ± 4.7	1.05 (0.91–1.21)	0.502
Gender (M)	6 (75.0%)	24 (49.0%)	3.13 (0.57–17.03)	0.188
ERM staging	2.29 ± 0.95	2.51 ± 0.77	0.68 (0.24–1.94)	0.478
BCVA	0.40 ± 0.18	0.47 ± 0.18	0.11 (0.00–6.07)	0.277
CMT (μm)	381 ± 72	452 ± 77	0.98 (0.97–1.00)	0.020
MV (mm^3^)	12.2 ± 1.8	11.8 ± 1.3	1.19 (0.70–2.01)	0.525
CME	2 (25.0%)	11 (22.5%)	1.15 (0.20–6.53)	0.873
IS/OS	1 (12.5%)	7 (14.3%)	0.86 (0.09–8.07)	0.893
NSD	2 (25.0%)	1 (2.0%)	16.0 (1.25–204)	0.033

## Discussion

**Fig. 1:**
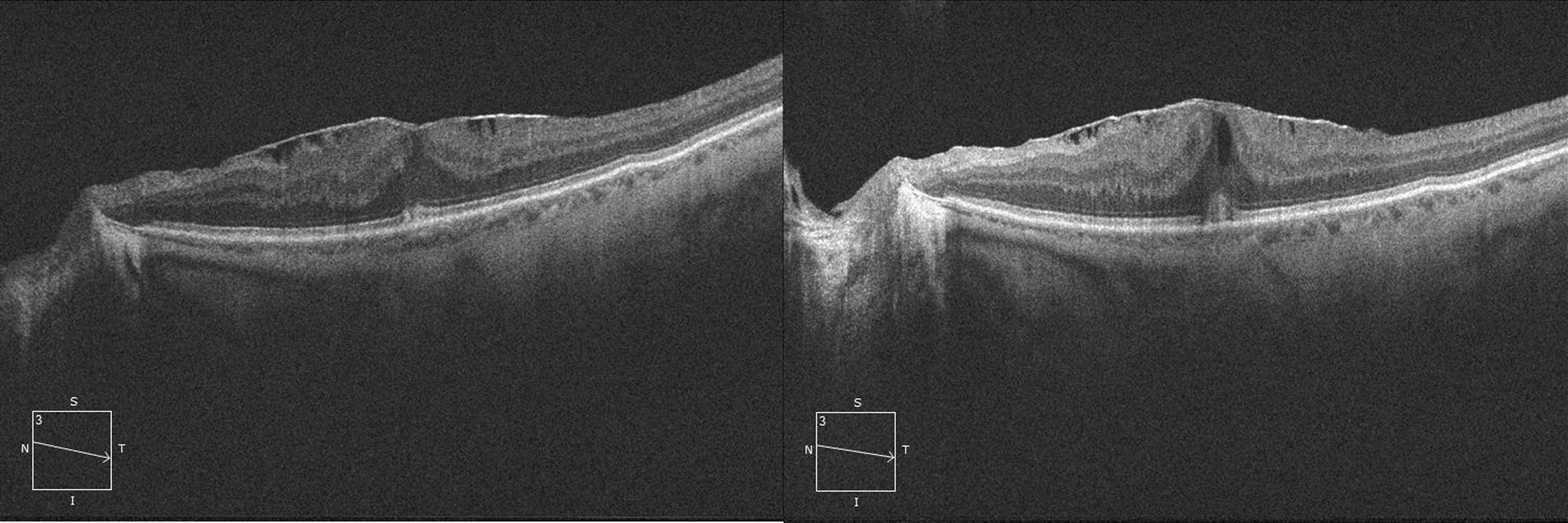
OCT showing a stage 2 ERM pre (left) and post (right) phacoemulsification. It can be observed a slight increase in the CME

Cataract surgery is a known factor causing inflammation of the eye. It has been described an upregulation of the inflammatory mediators in the aqueous and vitreous humors after surgical manipulation. The breakdown of the blood-aqueous and blood-retinal-barriers lead to an increased vascular permeability causing macular swelling with or without CME. Idiopathic epiretinal membranes are a result of a multifactorial activation of fibroblasts and the vitreous of these patients is known to have a higher concentration of inflammatory and profibrotic cytokines [9]. Chu et al. found that the relative risk of finding an increase in the relative risk of 5.60 in eyes with previously diagnosed ERM [10]. Our patient cohort demonstrated an incidence of postoperative CME at month 1 higher than what is reported in literature for patients with epiretinal membranes, which is around 15% [11]. Other studies [7] documented the presence or absence of ERM, not analyzing the stage of the condition, and we suggested that the advanced stages of our ERM could be responsible of this increased risk. However, there was no statistically significant correlation between the stage of the ERM and the development of the CME (Fig. 1).

A second aspect to consider is the improvement in the OCT technology, which allowed us to be way more sensitive in CME detection. According to a recently published paper by Dong H. Yoon et al., we classified as CME even the microcystic macular edema that is in fact a mild form of CME [12]. This increased sensitivity is confirmed by the fact that even preoperatively, one-fourth of our patients already had CME.

**Fig. 2:**
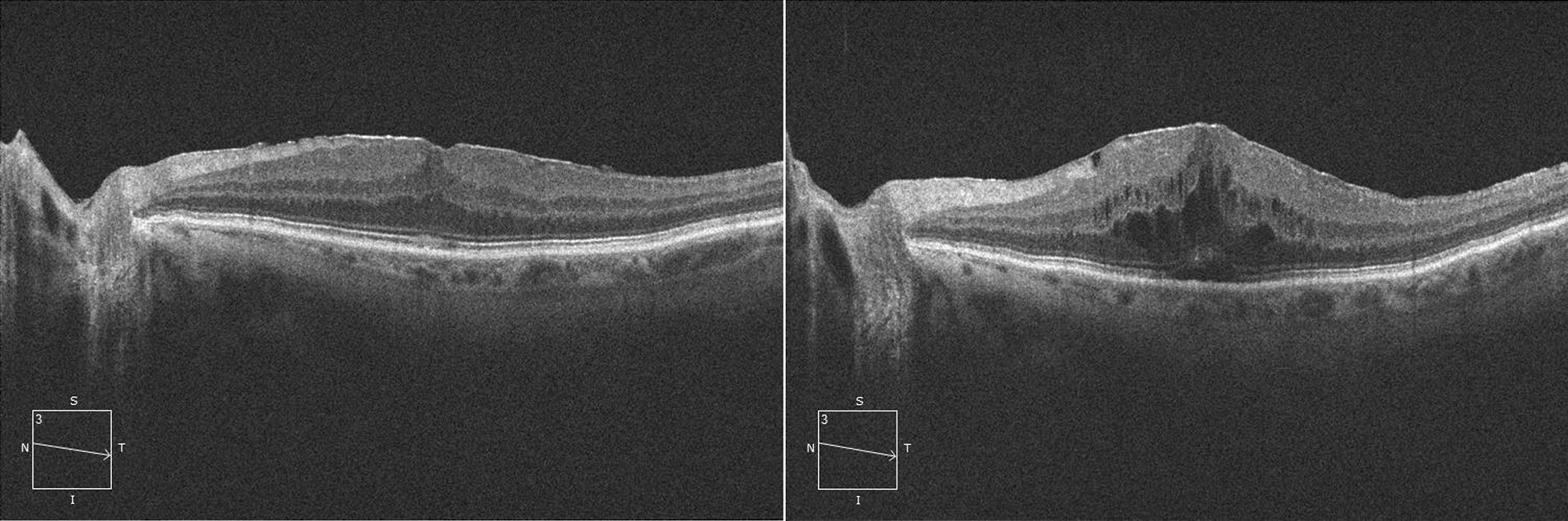
OCT showing a stage 3 ERM pre (left) and post (right) phacoemulsification. It can be observed a significant increase in the CME and a small subfoveal NSD

We also documented an increased rate of disruption of the inner/outer segment (IS/OS) and neuroretinal detachment (NSD) after phacoemulsification, all of them characteristics that are correlated to a reduced BCVA (Fig. 2).

Our data shows a statistically significant increase in all these variables. As literature has previously demostrated [7], the presence of an epiretinal membrane is an independent risk factor, which acts sinergically to increase the rate of these inflammatory consequences [13]. Detailed analysis of foveal OCT anatomy was performed demonstrating the changes induced by phacoemulsification in eyes with idiopathic advanced membranes.

There was no significant change in BCVA 1 month follow-up and we could not significantly correlate it to any specific foveal OCT changes. We acknowledge that one of the limitation of our study is that the BCVA does only partially reflect the inflammatory status of the ERM, given that a worsening of the retinal inflammatory status, might be compensated by BCVA improvement due to the cataract removal. However, most of our patient didn’t have a significant cataract, and the only indication to remove the lens was to perform a subsequent vitrectomy, so we might conclude that the cataract itself was not a cause for visual impairment.

We do not know whether this intraocular inflammation prolonged for longer follow up periods, would cause a worsening in the BCVA or resolve without consequences. In our case the maximum follow up was 3 months, because all our patients already had surgical ERM.

Previous studies have shown that the clinical history of an ERM is not significantly influenced by cataract surgery. Our findings confirm that even with this new OCT-based classification focused in the inner retinal changes the ERM progression is not significantly worsened by cataract surgery at 1 month follow up, however we are conscious that the short follow up is not enough to exclude the speed up of the natural history of ERM after phacoemulsification.

## Conclusion

Stage of the ERM was not rapidly accelerated by cataract surgery. Patients with ERM are at higher risk for developing inflammatory changes after cataract surgery such as cystoid macular edema, neurosensory detachment and alterations of the inner-outer segment layer. However, these are not associated with any worsening of the BCVA within the first month. We suggest that patients undergoing cataract surgery in the presence of epiretinal membranes need tight follow up to treat and control eventual macular inflammatory changes and eventual prompt vitrectomy if BCVA is threatened.

Further prospective studies are needed to fully understand the consequences of cataract surgery in patients with advanced epiretinal membranes.

## Data Availability

The data used to support the findings of this study are available from the corresponding author upon request.

## References

[1] Dikkaya F, Karaman Erdur S, Ozsutcu M, Aydin R, Kocabora MS, Aras C (2018). The significance of neutrophil-to-lymphocyte ratio in idiopathic epiretinal membrane. Int Ophthalmol.

[2] Xiao W, Chen X, Yan W, Zhu Z, He M (2017). Prevalence and risk factors of epiretinal membranes: a systematic review and meta-analysis of population-based studies. BMJ Open.

[3] Schumann RG, Gandorfer A, Ziada J (2014). Hyalocytes in idiopathic epiretinal membranes: a correlative light and electron microscopic study. Graefes Arch Clin Exp Ophthalmol.

[4] Hamoudi H, Correll Christensen U, La Cour M (2017). Epiretinal membrane surgery: an analysis of 2-step sequential- or combined phacovitrectomy surgery on refraction and macular anatomy in a prospective trial. Acta Ophthalmol.

[5] Dugas B, Ouled-Moussa R, Lafontaine PO (2010). Idiopathic epiretinal macular membrane and cataract extraction: combined versus consecutive surgery. Am J Ophthalmol.

[6] Alexandrakis G, Chaudhry NA, Flynn HWJ, Murray TG (1999). Combined cataract surgery, intraocular lens insertion, and vitrectomy in eyes with idiopathic epiretinal membrane. Ophthalmic Surg Lasers.

[7] Hayashi K, Hayashi H (2008). Influence of phacoemulsification surgery on progression of idiopathic epiretinal membrane. Eye.

[8] Govetto A, Lalane RA, Sarraf D, Figueroa MS, Hubschman JP (2017). Insights into epiretinal membranes: presence of ectopic inner foveal layers and a new optical coherence tomography staging scheme. Am J Ophthalmol.

[9] Zandi S, Tappeiner C, Pfister IB, Despont A, Rieben R, Garweg JG (2016). Vitreal cytokine profile differences between eyes with epiretinal membranes or macular holes. Invest Ophthalmol Vis Sci.

[10] Chu CJ, Johnston RL, Buscombe C, Sallam AB, Mohamed Q, Yang YC (2016). Risk factors and incidence of macular edema after cataract surgery: a database study of 81984 eyes. Ophthalmology.

[11] Schaub F, Adler W, Enders P (2018). Preexisting epiretinal membrane is associated with pseudophakic cystoid macular edema. Graefes Arch Clin Exp Ophthalmol.

[12] Yoon DH, Kang DJ, Kim MJ, Kim HK (2018). New observation of microcystic macular edema as a mild form of cystoid macular lesions after standard phacoemulsification. Medicine.

[13] Hardin JS, Gauldin DW, Soliman MK, Chu CJ, Yang YC, Sallam AB (2018). Cataract surgery outcomes in eyes with primary epiretinal membrane. JAMA Ophthalmol.

